# Outcomes of pediatric and adult patients with relapsed/refractory cortical (CD1a+) T-cell acute lymphoblastic leukemia. The Spanish experience from SEHOP and PETHEMA groups

**DOI:** 10.1007/s00277-026-06956-8

**Published:** 2026-03-24

**Authors:** Cristina Rivera-Pérez, Mireia Morgades, Anna Alonso-Saladrigues, Pau Montesinos, Thais Murciano, Cristina Gil, Rosa Adán, Jordi Esteve, Carolina Fuentes, María Luz Amigo, Berta González-Martínez, Rosa Coll, José Luis Dapena, María Paz Queipo de Llano, José Luis Fuster, Irene García-Cadenas, María Tasso, Pere Barba, Susana Rives, Josep Maria Ribera

**Affiliations:** 1 Stem Cell Transplant Department, Pediatric Cancer Center Barcelona (PCCB) - Hospital Sant Joan de Déu de Barcelona, Barcelona, Spain; 2 Faculty of Medicine, Universitat de Barcelona, Barcelona, Spain; 3 Clinical Hematology Department, Institut Català d’Oncologia - Hospital Germans Trias i Pujol, Badalona, Spain; 4 Leukemia and Lymphoma Department, Pediatric Cancer Center Barcelona (PCCB) - Hospital Sant Joan de Déu de Barcelona, Barcelona, Spain; 5 Institut de Recerca Sant Joan de Déu, Barcelona, Spain; 6 Hematology Department, Hospital Universitari i Politècnic La Fe, València, Spain; 7 Pediatric Oncology and Hematology Department, Hospital Universitari Vall d’Hebron, Barcelona, Spain; 8 Vall d’Hebron Research Institute (VHIR), Barcelona, Spain; 9 Hematology Department, Hospital General Universitari Dr. Balmis, Alacant, Spain; 10 Pediatric Hematology and Oncology Unit, Hospital Universitario de Cruces, Barakaldo, Spain; 11 Pediatric Oncology Research Group, Biobizkaia Health Research Institute, Barakaldo, Spain; 12 Hematology Department, Hospital Clínic de Barcelona, Barcelona, Spain; 13 Institut d’Investigacions Biomèdiques August Pi i Sunyer (IDIBAPS), Universitat de Barcelona, Barcelona, Spain; 14 Pediatric Oncology and Hematology Department, Hospital Universitari i Politècnic La Fe, València, Spain; 15 Hematology Department, Hospital General Universitario Morales Meseguer, Murcia, Spain; 16 Pediatric Hematology and Oncology Department, Hospital Universitario La Paz, Madrid, Spain; 17 Hematology Department, Institut Català d’Oncologia - Hospital Universitari Dr. Josep Trueta, Girona, Spain; 18 Hematology Department, Hospital Universitario Virgen de la Victoria, Málaga, Spain; 19 Hospital Clínico Universitario Virgen de la Arrixaca. El Palmar, Murcia, Spain; 20 Instituto Murciano de Investigación Biosanitaria (IMIB), El Palmar, Spain; 21 Hematology Department, Hospital de la Santa Creu i Sant Pau, Barcelona, Spain; 22 Pediatric Oncology and Hematology Department, Hospital General Universitari Dr. Balmis, Alacant, Spain; 23 Instituto de Investigación Sanitaria y Biomédica de Alicante (ISABIAL), Alacant, Spain; 24 Hematology Department, Hospital Universitari Vall d’Hebron, Barcelona, Spain; 25 Josep Carreras Leukemia Research Institute, Badalona, Spain; 26 Esplugues de Llobregat, Barcelona, 08950 Spain

**Keywords:** Relapsed/Refractory T-cell Acute Lymphoblastic Leukemia (R/R T-ALL), CD1a antigen, Survival, Immunotherapy

## Abstract

Patients with relapsed/refractory T-cell acute lymphoblastic leukemia (R/R T-ALL) have very poor prognosis. CAR T-cell therapy is being explored in these patients with encouraging results. Specifically, CD1a-directed CAR T-cells are being explored in clinical trials to treat cortical T-ALL (CD1a+), but there are no data on outcome in this subgroup of patients before this therapy. This retrospective, observational study aimed to describe the characteristics and outcomes of patients with R/R CD1a + T-ALL. We included pediatric and adult patients diagnosed with R/R CD1a + T-ALL in 25 sites of Spain between March 2006 and May 2022. The primary outcome of the study was overall survival (OS). Forty-three patients, 28 adults and 15 children, with R/R CD1a + T-ALL were included. Median (range) age at inclusion was 24 years (5–57), and 82.5% were male. After a median (range) follow-up of 7.0 years (5.1–13.6), 6 (14.0%) patients were alive and in complete remission (CR) and 37 (86.0%) had died. Five-year OS was 16% (95% CI; 7–29%). Bone marrow relapse, an interval < 12 months between first CR and relapse, and lack of allogeneic hematopoietic stem cell transplantation during salvage treatment were associated with worse OS. Our results confirm that patients with R/R CD1a + T-ALL have a very poor prognosis, highlighting the need for new treatment alternatives in these patients.

**Supplementary Information:**

The online version contains supplementary material available at 10.1007/s00277-026-06956-8.

## Introduction

T-cell acute lymphoblastic leukemia (T-ALL) is an aggressive hematological cancer originating from the malignant proliferation of T-cells. T-ALL accounts for 10% to 15% of pediatric ALLs and up to 25% of ALLs in adults [1, 2] and is classified into different subgroups based on immunophenotype. The most common subgroup corresponds to cortical T-ALL, which is characterized by the proliferation of leukemic cells surface expression of CD1a [3].

The prognosis for T-ALL is more favorable in children than in adults, since around 85–90% of pediatric patients can achieve cure [4], versus 40–50% of adult patients [5]. There are subgroups of patients with inferior outcome, such as those with early T-cell precursor ALL subtype [6–8]. Additionally, 15–20% of children and 40–50% of adults will relapse or be refractory to treatment, which results in a very poor prognosis, with a 5-year overall survival (OS) lower than 20–30% [9–11]. On the other hand, although the cortical T-ALL subtype at diagnosis carries the best prognosis, the prognosis of this subtype is unknown in relapsed/refractory (R/R) patients [5, 12].

There are currently several salvage treatment regimens for R/R T-ALL, including nelarabine, bortezomib, BCL2 inhibitors (venetoclax and navitoclax), daratumumab, among other drugs, but to date the only curative therapy is allogeneic hematopoietic stem cell transplantation (allo-HSCT) [13]. The CD1a antigen is currently targeted in Spain for chimeric antigen receptor T-cell (CAR-T) therapy in the setting of a clinical trial (EudraCT: 2024-514591-40-00). This clinical trial includes adult by pediatric patients with cortical T-ALL who are primarily refractory, with a first refractory relapse, in relapse after receiving an allo-HSCT or in a second or subsequent relapse. For this reason, the objective of this study was to analyze the survival of patients with R/R CD1a + T-ALL before the introduction of the aforementioned clinical trial.

## Methods

### Study design and patients

This was a retrospective, observational study of pediatric and adult patients diagnosed with R/R CD1a + T-ALL in 25 sites of Spain (Annex 1) between March 2006 and May 2022.

All clinical investigations were conducted according to the principles of the Declaration of Helsinki. The study was approved by the Institutional Review Board (IRB) of Hospital Sant Joan de Déu (PIC 98 − 22, available upon request) and Hospital Germans Trias i Pujol, which were the IRBs of reference for this study in Spain for pediatric and adult patients, respectively. Patients (or, alternatively, their legal representatives) signed the informed consent form before inclusion in the study.

### Variables and outcomes

Demographic and clinical variables included age, sex, leukocyte count, time interval from complete remission (CR) to relapse, localization of the relapse, treatments received and response. The primary outcome of the study was overall survival (OS), defined as the time interval from relapse or refractoriness to the death or last control.

### Statistical analysis

Quantitative variables were described as median and range, while categorical variables were presented as frequencies and percentages. OS was calculated by means of the Kaplan-Meier method. To estimate the effect on OS of allo-HSCT during salvage treatment, the transplantation variable was considered as a time-dependent covariable within the Cox model, and the OS plot was represented by means of the Simon-Makuch method. The analysis of prognostic factors for OS was performed by means of Cox’s proportional hazards model, considering in the multivariable analysis those variables with *P* < 0.100 in the univariate analysis except when collinearity between variables was identified. The level of statistical significance was established at a bilateral alpha level of 0.05. All statistical analyses were made using the SPSS v.24 and R v.4.2.0 software.

## Results

### Characteristics of patients

Out of 110 patients with R/R T-ALL, 43 (28 adults and 15 children) expressed CD1a and were included in the analysis. Overall survival of CD1a positive patients did not differ from that of those CD1a negative (Figure S1). The characteristics of CD1a positive patients at diagnosis are shown in Table 1. Three patients (7.0%) were primarily refractory, and 40 (93.0%) had experienced relapse.

**Table 1:** Characteristics of study patients at diagnosis, *n* = 43^a^

Demographic characteristics	
Age (years), *median (range)*	24 (4–56)
Male, *n (%)*	35 (81.4)
Median time of relapse (months)	12 (1–32)
Clinical characteristics	
Extramedullary involvement, *n (%)*	
Nodal (*n* = 40)	25 (62.5)
Hepatic (*n* = 39)	16 (41.0)
Splenic (*n* = 41)	21 (51.2)
Mediastinal	21 (48.8)
CNS	10 (23.2)
Other	5 (11.6)
White blood cell count (x10^9^/L), *median (range)*	75.0 (1.4–456.4)

^a^unless otherwise indicated. *CNS* central nervous system

Of 28 adult patients, 14 (50.0%) had received first-line treatment according to the protocol of the Spanish Hematology Treatment Program (*Programa Español de Tratamientos en Hematología*, PETHEMA) ALL-AR-03 [14] and 14 (50.0%) according to the PETHEMA protocol ALL-AR-11 [15]. All pediatric patients (*n* = 15, 100%) had received first-line treatment according to the recommendations of the Spanish Pediatric Hematology and Oncology Society (*Sociedad Española de Hematología y Oncología Pediátricas*, SEHOP-2005) [4] and LAL/SEHOP-PETHEMA 2013 [16].

The median age (range) at relapse was 24 (5–57) years. Twenty patients relapsed before 12 months from diagnosis, and 25 had a bone marrow relapse (isolated or combined). The central nervous system (CNS) was the most prevalent extramedullary localization, affecting 18 patients (Table 2).

**Table 2:** Characteristics of the study patients at the time of first relapse. *N*=40^a^

Age (years), median (range)	24 (5–57)
Male, *n (%)*	33 (82.5)
Time from first complete remission to relapse	
Median (range), months	12 (1–32)
< 12 months	20 (50.0)
≥ 12 months	20 (50.0)
Relapse site, *n (%)*	
Medullary	14 (35.0)
Extramedullary	15 (37.5)
Combined^b^	11 (27.5)
Extramedullary involvement^c, d^, *n (%)*	
CNS	18 (45.0)
Isolated	11 (27.5)
Combined	7 (17.5)
Mediastinal	3 (7.5)
Cutaneous	2 (5.0)
Testicular	2 (5.0)
Lymph nodes	1 (2.5)
Pericardial	1 (2.5)
Pleural	1 (2.5)
Multiple organs	1 (2.5)

^a^ Data were excluded for three patients who were primarily refractory. ^b^ Medullary and extramedullary involvement. ^c^ Includes isolated and combined extramedullary involvement. ^d^ More than one option is possible. *CNS* central nervous system.

**Fig. 1:**
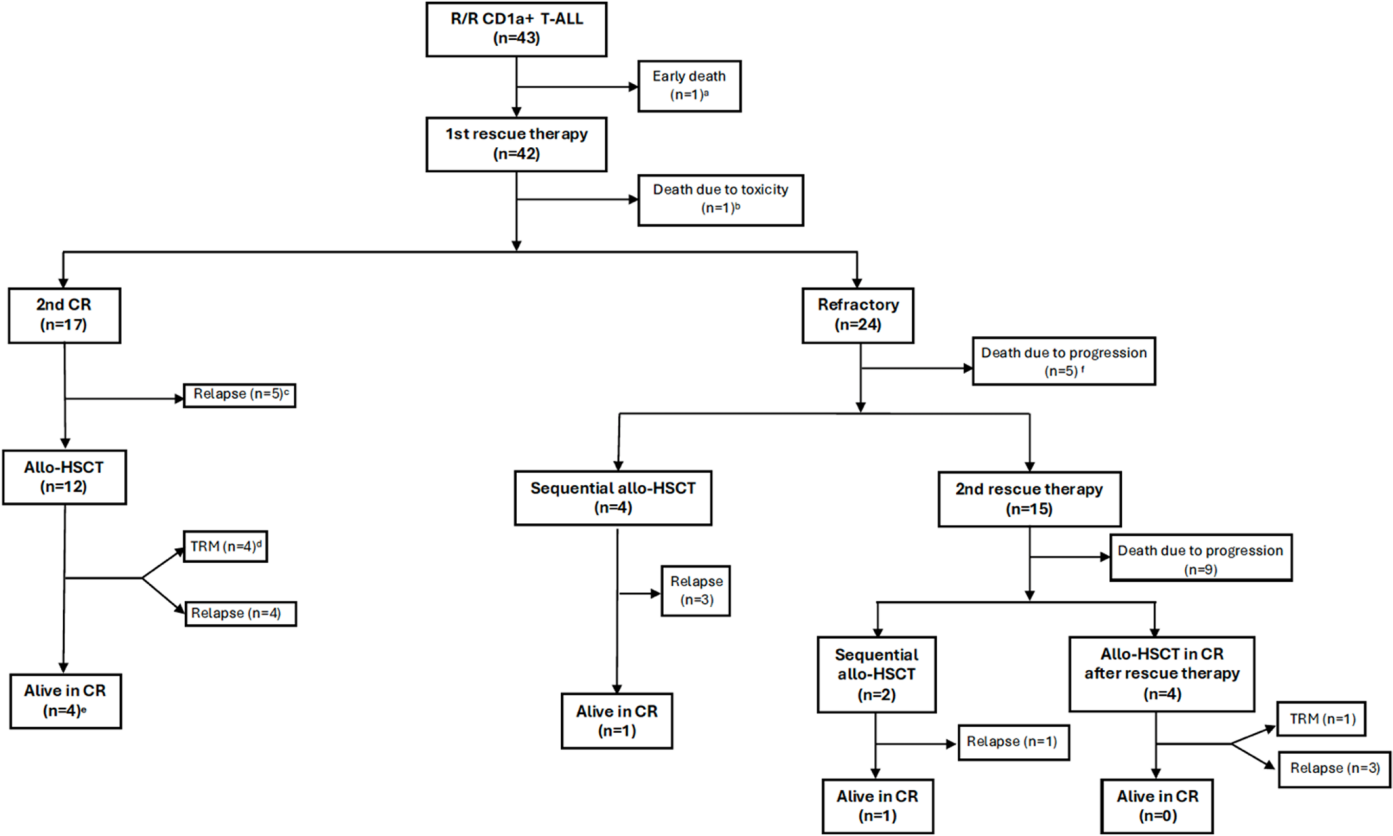
Treatment response and outcomes of study patients. 14 patients excluded as eligible for CAR-T therapy in this analysis: ^a^ 1 patient for early death, ^b^ 1 patient who died due to toxicity during first line of relapse treatment ^c^ 1 patient due to psychiatric disease (the other four patients would be eligible), ^d^ 4 non-eligible according to inclusion criteria, ^e^ 4 non-eligible according to inclusion criteria, ^f^ 3 non-eligible patients due to early death (by progression) in first rescue treatment (the other two would be eligible: one of them was a post Allo-HSCT relapse and the other one double refractory)

Of the 43 patients, 2 (4.7%) had received an allogeneic HSCT in first complete remission (CR), 4 had received an allogeneic HSCT in CR-2, 3 in CR-3 and 4 received a sequential allogeneic HSCT after receiving 1 or 2 salvage therapy lines (Fig. 1). All patients with post‑transplant relapse were included in the survival analyses, both in the overall cohort (*n* = 43) and in the subgroup considered eligible for CAR‑T therapy.

### Treatment response and outcomes

Figure 1 shows the rescue treatments, the response and outcome of the patients. One patient died on the same day of diagnosis of the first relapse, and the remaining 42 patients received first-line salvage treatment, with FLAG-IDA (fludarabine, cytarabine, granulocyte-colony-stimulating factor, and idarubicin) being the most common. One patient (2.4%) died, 17 (40.5%) patients achieved a second CR, and 24 (57.1%) were refractory to treatment. Of the 41 patients in second CR or refractory, 21 (51.2%) received a second line of treatment, with nelarabine and methotrexate being the most frequently used drugs (Table S1).

Of the 17 patients who reached a second CR, 12 (70.6%) received an allo-HSCT as treatment consolidation, after which 4 (33.3%) patients died following a new relapse, 4 (33.3%) died due to transplant-related toxicity, and 4 (33.3%) were still alive in CR. The remaining patients (*n* = 5, 29.4%), who did not receive an allo-HSCT as treatment consolidation, experienced an early relapse (median [range] of 57 [36–95] days). Of the 24 patients who were refractory to the first salvage treatment, 10 (41.7%) received an allo-HSCT (4 in second CR), after which 7 (70.0%) patients died after a new relapse, 1 (10.0%) died due to transplant-related toxicity and 2 (20.0%) were still alive in CR (Fig. 1).

### Characteristics of patients potentially eligible for CD1a CAR-T therapy

Of all the study patients, 29 would have been eligible to receive CD1a directed CAR-T therapy: 1 primarily refractory patient and 28 relapsed patients (Fig. 1). The characteristics of the 28 patients who were potentially eligible to receive CAR-T therapy are summarized in Table 3. The median (range) age at relapse was 22 (5–57) years. Sixteen patients relapsed before 12 months of the first CR and 21 experienced a bone marrow relapse (isolated or combined). The most common extramedullary localization was the CNS, affecting almost 40% of the patients.

**Table 3:** Characteristics of relapsed or refractory patients after two or more lines of treatment. *N*=28a

Demographic	
Age (years), *median (range)*	22 (5–57)
Male, *n (%)*	23 (82.1)
Clinics	
Time from first complete remission to relapse	
Median (range), months	10 (1–32)
< 12 months	16 (57.1)
≥ 12 months	12 (42.9)
Relapse site ^b^, *n (%)*	
Medullary	11 (39.3)
Extramedullary	7 (25.0)
Combined	10 (35.7)
Extramedullary involvement^c, d^, *n (%)*	
CNS	11 (39.3)
Isolated	5 (17.9)
Combined	6 (21.4)
Mediastinal	3 (10.7)
Cutaneous	2 (7.1)
Testicular	1 (3.6)
Lymph nodes	1 (3.6)
Pericardial	1 (3.6)
Pleural	1 (3.6)

^a^ Data were excluded for one patient who was primarily refractory. ^b^ Medullary and extramedullary involvement. ^c^ Includes isolated and combined extramedullary involvement. ^d^ More than one option is possible. *CAR-T* chimeric antigen receptor T-cell, *CNS* central nervous system.

A subsequent investigation revealed no statistically significant differences between the CAR-T eligible and the non-eligible cohorts in terms of the median time to relapse (10 months vs. 13 months, respectively, see Table S2), nor were there any differences in the number of allo-HSCT received between the CAR-T eligible and the general cohorts (15/29 vs. 23/43). However, patients who would have met eligibility criteria for CAR-T therapy had a lower number of isolated extramedullary relapses (*p* = 0.038).

### Overall survival

After a median (range) follow-up of 7.0 years (5.1–13.6), 6 (14.0%) patients were still alive and in CR, while the remaining 37 (86.0%) had died. The main cause of death was progression of the disease (*n* = 29, 78.4%), followed by transplant-related mortality (TRM) (*n* = 7, 18.9%) and infection during salvage treatment (*n* = 1, 2.7%). No differences in the OS over the study period were observed.

**Fig. 2:**
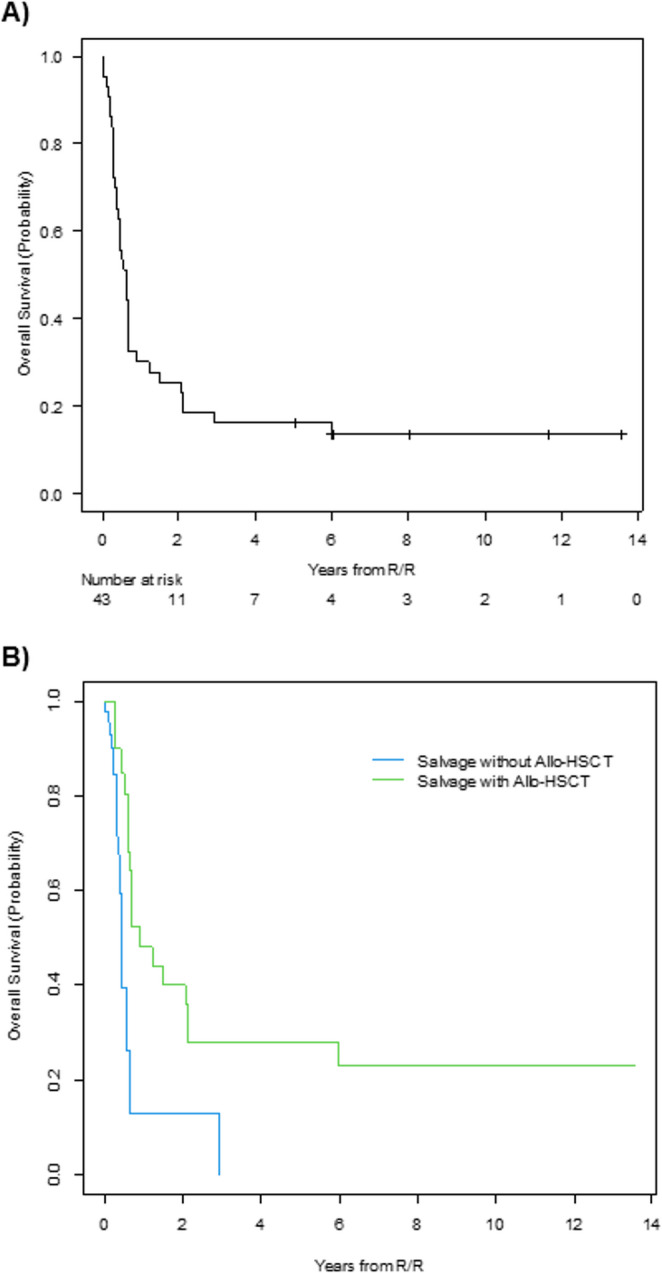
Overall survival of all study patients (A) and of all the patients who received a first salvage treatment, based on presence or absence of an allogeneic hematopoietic stem cell transplantation (allo-HSCT) (B)

The median OS of the series was 7.3 months (95% CI; 4.4–8.2) and the 5-year OS was 16% (95% CI; 7–29%) (Fig. 2A). Patients who did not receive an allo-HSCT at any time during salvage treatment showed a higher risk of death than those who underwent an allo-HSCT (HR 2.9 [95% CI; 1.2–6.9], *p* = 0.015) (Fig. 2B).

**Fig. 3:**
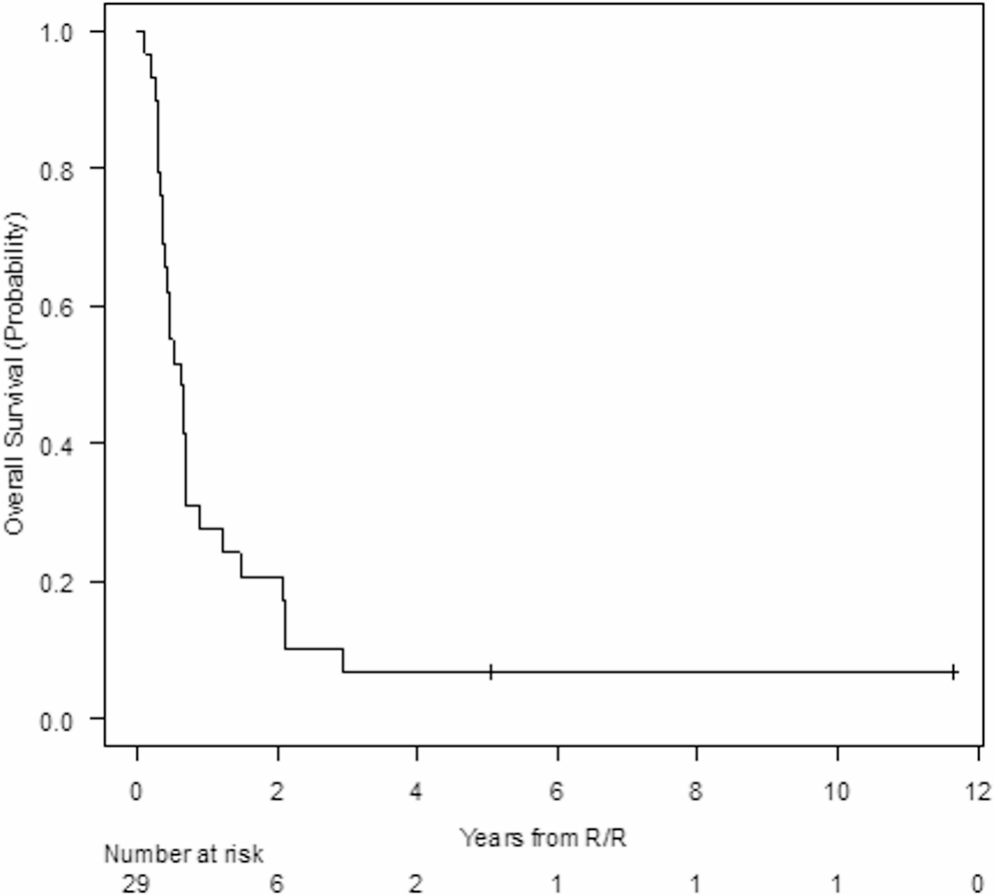
Overall survival of the subgroup of patients who are potential eligible to receive CD1a CAR-T therapy

As regards the subgroup of 29 patients who were eligible to receive CD1a directed CAR-T therapy, the median OS in this subgroup of patients was 7.5 (95% CI; 4.5–8.2) months and the 5-year OS was 7% (95% CI; 1–20%) (Fig. 3). At the time of data cutoff, only one patient is alive and in CR.

### Prognostic factors for overall survival

Bone marrow relapse and refractoriness to the first line of salvage treatment were associated with a poorer OS in the univariate analysis. In the multivariable analysis, bone marrow relapse (isolated or combined), an interval inferior to 12 months between first complete remission and relapse, and absence of an allo-HSCT during salvage treatment were also associated with a poor OS (Table 4). Five years OS stratified by age group was 13% (95% CI: 2%–35%) in pediatric patients and 18% (95% CI: 2%–35%) in adults.

**Table 4:** Prognostic factors for overall survival based on univariate and multivariable analysis

Factor	Univariate analysis		Multivariable analysis	
	HR (95% CI)	*P*	HR (95% CI)	*P*
Age at relapse	0.983 (0.959–1.008)	0.176		
Age at relapse				
≤ 18 years	1.183 (0.570–2.455)	0.653		
> 18 years	Reference			
Relapse localization				
Bone marrow ± extramedullary	2.560 (1.188–5.516)	0.016	3.199 (1.336–7.656)	0.009
Extramedullary	Reference		Reference	
Isolated CNS involvement at relapse				
Yes	Reference	0.006		
No	3.632 (1.455–9.069)			
Time from 1st complete remission to relapse				
< 12 months	1.876 (0.935–3.761)	0.076	2.149 (1.035–4.464)	0.04
≥ 12 months	Reference		Reference	
Response to first salvage regimen				
Complete remission	Reference	0.018		
Failure/ED	2.456 (1.167–5.167)			
Total number of salvage regimens				
1	Reference	0.159		
≥ 2	1.682 (0.815–3.469)			
Allo-HSCT during salvage treatment				
Yes	Reference	0.057	Reference	0.021
No	2.468 (0.975–6.254)		3.798 (1.228–11.748)	

*Allo-HSCT* allogeneic stem cell transplantation, *ED* early death, *CNS* central nervous system

## Discussion

This retrospective study of patients with R/R CD1a + T-ALL confirmed that the poor prognosis for patients with R/R T-ALL is also observed in the sub-group of patients with cortical T-ALL. Five-year OS in our series was very similar to that of adult patients with all T-ALL subtypes in Spain [10]. However, it was lower than that described for other groups in relapsed pediatric patients with T-ALL [11]. The lower OS in our series could be due to the heterogeneity of the second-line treatments together to the lack of clinical trials with new drugs for patients with relapsed T-ALL in the study period.

The low number of patients and the diversity of treatments employed in second and subsequent rescue lines hinder the assessment of reliable prognostic factors in the relapse. However, and similarly to the results obtained in other series of patients with relapsed ALL, the multivariable analysis showed that isolated extramedullary relapse and a longer duration of the first CR were associated with a better survival [10, 17, 18]. Additionally, we observed that the lack of performance of allo-HSCT was an additional poor prognosis factor, as previously described by other authors [11, 18]. Finally, and consistently with previous studies [10, 17], the prognosis was even poorer for patients who were refractory to the first salvage treatment and especially for those patients who would have been eligible for CAR-T cell therapy.

The special poor prognosis for patients who are refractory to first salvage treatment indicates that there is a critical need to find new treatments in this subgroup of patients. Among them, special mention should be made to combinations of chemotherapy agents [19] such as nelarabine [20] and targeted therapies like proteasome inhibitors (e.g., bortezomib) [21] or BCL-2 inhibitors, like venetoclax [22]. In addition, similarly to B-cell ALL (B-ALL), immunotherapy is being explored for the treatment of T-ALL, using monoclonal antibodies like daratumumab [23–25] or CAR-T cell therapy.

CAR-T cell therapy has proven effective in patients with B-ALL [26]. However, is less developed for T-ALL, due to the inherent difficulty of targeting T-cells, which include potential CAR-T cell fratricide [27]; potential contamination with blasts resulting from apheresis and subsequent insertion of the CAR in T blast cells; and T cell aplasia, which could render the patient severely immunocompromised [28]. In this regard, different targets have been investigated for CAR-T cells in T-ALL (e.g., CD7, CD5, CD1a and CD38), and several techniques have been developed to avoid the difficulties described above [29–33]. Thus, CAR-T therapy against CD1a could be a good alternative, which is being studied in Phase I clinical trials in Spain (NCT05679895) and China (NCT05745181**)**.

The main limitations of this study are its retrospective nature, the limited number of patients, the lack of genetic and molecular characterization and the heterogeneity of the rescue treatments (including first- and second-line salvage therapies, which includes several chemotherapy regimens, radiotherapy and different targeted therapies). However, it provides the real-world results of patients with R/R cortical T-ALL treated in Spain during the study period. These results could be useful in the future when the results of the currently ongoing clinical trial in patients treated with CAR-T against CD1a become available.

In summary, this study has shown that patients with R/R CD1a + T-ALL had a very poor prognosis, with a 5-year OS rate lower than 20% in first relapse and lower than 10% in the cases of a second relapse or refractoriness to two or more lines of treatment. This very poor prognosis highlights the need for new and more effective rescue treatments for these patients.

## Supplementary Information

Below is the link to the electronic supplementary material.

Supplementary Material 1

## Data Availability

The data will be made available from request to researchers who provide a methodologically sound proposal. The data will be provided after de-identification, in compliance with applicable privacy and data protection laws, and requirements for consent and anonymization.

## Appendix

### Study participating sites and number of patients included at each site

Hospital La Fe (Valencia) 6, Hospital Vall d’Hebron (Barcelona) 5, Hospital General Alicante (Alicante) 4, Hospital Clínic de Barcelona (Barcelona) 2, Hospital Morales Meseguer (Murcia) 2, ICO-Hospital Josep Trueta (Girona) 2, Hospital Clínico Universitario Virgen de la Victoria (Málaga) 2, Hospital Cruces – Baracaldo (Bilbao) 2, Hospital la Paz (Madrid) 2, Hospital Sant Pau (Barcelona) 1, Hospital Virgen del Rocío (Sevilla) 1, Hospital Clínico Valencia (Valencia) 1, Hospital Gregorio Marañón (Madrid) 1, Hospital Lucus Augusti (Lugo) 1, Hospital Universitario de Canarias (Tenerife) 1, Hospital de Pontevedra (Pontevedra) 1, Hospital General Universitario de Albacete (Albacete) 1, Hospital Universitario de León (León) 1, Hospital Universitario Santiago Compostela (Santiago) 1, ICO-Hospital Duran i Reynals (Barcelona) 1, Hospital Carlos Ayala (Málaga) 1, Hospital Universitario Son Espases (Mallorca) 1, Hospital Universitario Niño Jesús (Madrid) 1, Hospital Parc Taulí (Sabadell) 1, Hospital Universitario Jaén (Jaen) 1.
