# Supplementary material for: Outcomes of pediatric and adult patients with relapsed/refractory cortical (CD1a+) T-cell acute lymphoblastic leukemia. The Spanish experience from SEHOP and PETHEMA groups
Source: Ann Hematol. 2026 Mar 24;105(4):203. doi: 10.1007/s00277-026-06956-8 (PMC13013216; doi:10.1007/s00277-026-06956-8)
Supplement: Supplementary file 1 — Supplementary Material 1 [file 277_2026_6956_MOESM1_ESM.docx]

**Supplementary materials for the submission “**Outcomes of Pediatric and Adult patients with Relapsed/Refractory Cortical (CD1a+) T-cell Acute Lymphoblastic Leukemia. The Spanish experience from SEHOP and PETHEMA groups” by Rivera-Pérez *et al*. to the journal Annals of Hematology.

Authors:

Cristina Rivera-Pérez, Mireia Morgades, Anna Alonso-Saladrigues, Pau Montesinos, Thais Murciano, Cristina Gil, Rosa Adán, Jordi Esteve, Carolina Fuentes, María Luz Amigo, Berta González-Martínez, Rosa Coll, José Luis Dapena, María Paz Queipo de Llano, José Luis Fuster, Irene García-Cadenas, María Tasso, Pere Barba, Susana Rives, Josep Maria Ribera.

Corresponding author:

Susana Rives

Leukemia and Lymphoma Department. Pediatric Cancer Center Barcelona (PCCB) - Hospital Sant Joan de Déu de Barcelona. Barcelona, Spain

Institut de Recerca Sant Joan de Déu. Barcelona, Spain.

e-mail: [susana.rives@sjd.es](mailto:susana.rives@sjd.es)

**Figure S1**. Overall survival between CD1a‑negative and CD1a‑positive subgroups.


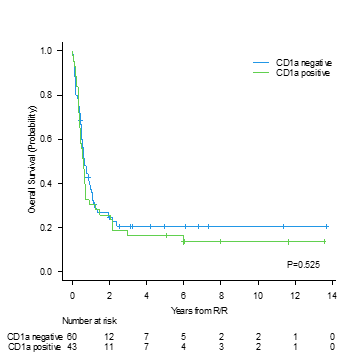


| **Table S1.** First- and second-line salvage treatments. *N (%).* | |
| --- | --- |
|  |  |
| **First line (N=42)** |  |
| FLAG-IDA ±TIT | 18 (42.9) |
| Nelarabine ± Others | 5 (11.9) |
| HyperCVAD | 2 (4.8) |
| Clofarabine ± Others | 2 (4.8) |
| Others | 15 (35.7) |
| **Second line (N=21)** |  |
| Nelarabine ± Otros | 7 (33.3) |
| MTX ± Citarabine ± TIT | 5 (23.8) |
| Radiotherapy | 3 (14.3) |
| HyperCVAD | 2 (9.5) |
| FLAG-IDA | 1 (4.8) |
| Others | 3 (14.3) |
| FLAG-IDA, fludarabine, cytarabine, granulocyte-colony-stimulating factor, and idarubicin; HyperCVAD, cyclophosphamide, vincristine, doxorubicin, dexamethasone; MTX, methotrexate; TIT, triple intrathecal therapy. | |
|  |  |
|  |  |
|  |  |

| **Table S2.** Characteristics of patients who are candidates and non-candidates for CAR-T cd1a therapy | | | |
| --- | --- | --- | --- |
|  | Eligible to CAR-T (n=29) | Not eligible to CAR-T (n=14) | *P* |
| Age (yr), median (range) | 23 (4-56) | 33 (7 -45) | 0.414 |
| Male, n (%) | 24 (83%) | 11 (79%) | >0.999 |
| Median time of relapse (months) | 10 (1 – 32) | 13 (2 – 31) | 0.894 |
| Relapse localization   - BM+extramedullary - Extramedullary | 21/28 (75%)  7/28 (25%) | 5/13 (38.5%)  8/13 (61.5%) | 0.038 |
| WBC, median (range) | 75 (1.4 – 399.6) | 88.4 (4.9 – 456.4) | 0.351 |
| Allo-HSCT performed | 15 (52%) | 8 (57%) | 0.739 |
| BM: bone marrow. WBC: white blood counts. HSCT: hematopoietic stem cell transplantation | | | |
